# Can an Abusive Supervision Be a Predictor of Doocing? Comment on Akram, Z.; Li, Y.; Akram, U. When Employees Are Emotionally Exhausted Due to Abusive Supervision. A Conservation-of-Resources Perspective. *Int. J. Environ. Res. Public Health* 2019, *16*, 3300

**DOI:** 10.3390/ijerph17249370

**Published:** 2020-12-15

**Authors:** Stefania Fantinelli

**Affiliations:** Department of Psychological, Health and Territory Sciences, Università “G. d’Annunzio” of Chieti-Pescara, 66100 Chieti, Italy; stefania.fantinelli@unich.it

**Keywords:** abusive supervision, counterproductive work behavior, doocing

## Abstract

Thanks to the research work of Akram and colleagues on the consequences of an abusive supervision, it is possible to hypothesize a new point of view of the doocing phenomenon. According to the authors, an abusive supervision can cause, through the interaction of some mediators and moderators, counterproductive work behaviors; this comment proposes that these behaviors can be performed also in an online context. As a consequence, a worker could be fired because of something posted on social media (doocing). Another relevant point of view concerns the great responsibility given to supervisors and management with regard to the care of job environment from an emotional point of view.

The work of Akram and colleagues is focused on the counterproductive work behaviors (CWBs), meant as “activities that are unhealthy for the effectiveness of the organization”, the primary aim is to investigate how abusive supervision can impact such deviant behaviors [1]. An abusive supervision can also be responsible for a state of employees’ emotional exhaustion that can, in turn, cause deviant behaviors. Workers who feel emotionally exhausted perceive more job emotional demands, so that, when job demands are high, the relationship between abusive supervision and emotional exhaustion is stronger.

The theoretical frame of the study is the conservation of resources theory: one of the main objective for individuals is to preserve valuable resources such as energy and time [2]. So that employees who perceive threats to their resources, because of abusive behaviors, tend to experience emotional exhaustion that can contribute to the involvement in counterproductive work behaviors. Indeed, the main hypothesis of the study is confirmed: Akram and colleagues find a positive relationship between abusive supervision and counterproductive work behaviors.

It can be stated that counterproductive work behaviors can also concern online behaviors; as stated by Floridi [3] we are living in an *onlife* dimension, we are always connected: there is not a precise border between what we do in a real face-to-face contexts and what we do in a digital dimension, such as social media. In line with this point of view, we consider that a possible emotional outburst on social media related to a discomfort or to a job stressor could represent a sort of counterproductive work behavior.

Indeed, when a worker on social media talks about colleagues or supervisors or the organization in bad terms or expressing dislike, the online reputation of the entire organization can be damaged. This behavior and its possible consequence can be framed in terms of *doocing*, which is defined as job termination caused by illicit or inappropriate behavior on social media that does not fit with the corporate policies [4,5,6].

If we frame online deviant work behaviors as a predictor of doocing together with a bad supervision, we have a new point of view for the interpretation of the phenomenon of doocing: a worker who posts an disrespectful comment about the employer or the organization could also have a reason to do it, a sort of answer to an unfair treatment, such as an abusive supervision. This statement is in line with a principle of the pragmatics of human communication [7]: the different framing of an event. In this sense the same behavior can be interpreted as a stimulus for someone and as a reaction for someone else. Indeed, researchers confirmed that a lack of social support and well-being can encourage workers to engage in counterproductive behaviors; interpreting the doocing phenomenon in this sense means to give great responsibility to a supervisor, in particular for what concerns the employees’ mood and the work emotional context.

With regard to those variables, it can be relevant to include the constructs emotional labor and trait emotional intelligence to improve our understanding of individual differences in experiencing the risk of feeling emotionally exhausted under the same circumstances. More specifically, emotional labor is strictly related to those work conditions requiring high level of interpersonal relations and it implies the management of emotions in line with organizational display rules [8]. When an employee chooses “surface acting” there is only a modification of external expression, but it is an unsuccessful strategy since it can increase emotional dissonance and it is also associated with burnout and depression. High scores on trait emotional intelligence can help individuals to experience more positive emotions [8], on the other side, a low level of trait emotional intelligence can represent a predictor of counterproductive work behaviors. There are several interpretations of the construct of trait emotional intelligence (EI), summarizing them, EI can be both a personality trait in its noncognitive component and an ability as a type of intelligence in its conscious factor [9].

Future researches should contemplate including the traits of emotional intelligence and emotional labor as mediator factors in those models predicting the risk of doocing.

This is not to underestimate the need for a social media use training, which should be provided by organizations for all the employees [6,10]; in order to increase the awareness on the themes of online privacy and reputation management.

An interesting strength of this study is the enrollment of both employees and supervisors, totalling 350 supervisor-subordinate dyads; there are several practical and theoretical implications to highlight. As suggested by the authors, supervisors should be monitored in order to observe, and limit or avoid, an abusive supervision; so a strong practical implication regards the supervision of both employees and supervisors, with a specific training for the latter, considering that a supervisor should represent a sort of role model for workers. The authors also depicted some limitations of the study: it was not a longitudinal study, so it was not possible to establish causality; data were collected from only one job sector and only in China, so a cross-cultural study and other job sectors is a recommendation for the future.

Moreover, it can be said that there are several dimensions to take into account in order to keep a healthy and happy work environment and to avoid counterproductive behaviors; one possible action can be a training for gratitude, surely something very far from an abusive supervision. Past researches highlighted how the collective gratitude of employees toward the organization can contribute to enhance workers well-being [11], other than foster both job performance and job satisfaction [12].
